# Patients with NPSLE experience poorer HRQoL and more fatigue than SLE patients with no neuropsychiatric involvement, irrespective of neuropsychiatric activity

**DOI:** 10.1093/rheumatology/keae216

**Published:** 2024-04-05

**Authors:** Dionysis Nikolopoulos, Nursen Cetrez, Julius Lindblom, Leonardo Palazzo, Yvonne Enman, Ioannis Parodis

**Affiliations:** Division of Rheumatology, Department of Medicine Solna, Karolinska Institutet, Stockholm, Sweden; Department of Gastroenterology, Dermatology and Rheumatology, Karolinska University Hospital, Stockholm, Sweden; Division of Rheumatology, Department of Medicine Solna, Karolinska Institutet, Stockholm, Sweden; Department of Gastroenterology, Dermatology and Rheumatology, Karolinska University Hospital, Stockholm, Sweden; Division of Rheumatology, Department of Medicine Solna, Karolinska Institutet, Stockholm, Sweden; Department of Gastroenterology, Dermatology and Rheumatology, Karolinska University Hospital, Stockholm, Sweden; Division of Rheumatology, Department of Medicine Solna, Karolinska Institutet, Stockholm, Sweden; Department of Gastroenterology, Dermatology and Rheumatology, Karolinska University Hospital, Stockholm, Sweden; Division of Rheumatology, Department of Medicine Solna, Karolinska Institutet, Stockholm, Sweden; Department of Gastroenterology, Dermatology and Rheumatology, Karolinska University Hospital, Stockholm, Sweden; Division of Rheumatology, Department of Medicine Solna, Karolinska Institutet, Stockholm, Sweden; Department of Gastroenterology, Dermatology and Rheumatology, Karolinska University Hospital, Stockholm, Sweden; Faculty of Medicine and Health, Department of Rheumatology, Örebro University, Örebro, Sweden

**Keywords:** systemic lupus erythematosus, CNS lupus, patient-reported outcomes, quality of life

## Abstract

**Objectives:**

Substantial proportions of patients with SLE report poor health-related quality of life (HRQoL). Our objective was to investigate the impact of neuropsychiatric involvement (NP) in SLE on patient-reported outcomes.

**Methods:**

We analysed data from four phase III trials (BLISS-52, BLISS-76, BLISS-SC, EMBRACE; *N* = 2968). The NPSLE group comprised individuals with NP-BILAG A/B/C/D or score in any descriptor of the NP-SLEDAI-2K at baseline (*N* = 350), while the non-NPSLE group consisted of patients with NP-BILAG E (*N* = 2618). HRQoL was assessed with the SF-36, EQ-5D-3L, and FACIT-F. Full health state (FHS) was defined as ‘no problems’ in all EQ-5D dimensions.

**Results:**

NPSLE patients reported lower scores in the SF-36 physical and mental component summary compared with the non-NPSLE population [mean (s.d.): 35.7 (9.1) *vs* 39.6 (9.6); *P* *<* 0.001 and 37.3 (12.1) *vs* 41.4 (11.0); *P* *<* 0.001, respectively]. NPSLE patients also exhibited impaired HRQoL in all EQ-5D dimensions compared with non-NPSLE patients (*P* *<* 0.05 for all). A substantially lower proportion of NPSLE patients experienced FHS in comparison with the non-NPSLE group (3.3% *vs* 14.5%; *P* *<* 0.001). NPSLE was associated with severe fatigue [23.8 (12.2) *vs* 31.5 (11.6); *P* *<* 0.001]. Notably, our findings revealed no discernible distinctions between active and inactive NPSLE patients with regard to SF-36, EQ-5D, FHS or FACIT-F scores.

**Conclusion:**

NP in patients with SLE has a detrimental effect on HRQoL experience and is associated with severe fatigue, regardless of the degree of neuropsychiatric disease activity. Early intervention is warranted in NPSLE patients to enhance long-term HRQoL experience.

Rheumatology key messages

- Patients with NPSLE experience worse HRQoL than SLE patients with no neuropsychiatric disease.
- NPSLE is associated with greater fatigue levels compared with SLE without neuropsychiatric involvement.
- HRQoL impairments in patients with NPSLE persist despite resolution of the neuropsychiatric activity.

## Introduction

Approximately 20% of patients with SLE experience primary neuropsychiatric events, collectively termed neuropsychiatric SLE (NPSLE) [1]. Defining the outcome of NPSLE poses challenges due to the wide range of clinical syndromes and associated neurological deficits [2]. Increased rates of morbidity and mortality have been associated with neuropsychiatric involvement in lupus [3, 4], yet our knowledge of the effects of NPSLE on patient-reported outcomes (PROs) is limited. While most neuropsychiatric events are resolved in SLE [4], recent studies suggest a negative impact on patients’ health-related quality of life (HRQoL) [5]. A comprehensive characterization of PROs in patients with NPSLE is currently lacking.

Over the past few decades, there has been an increasing recognition of the importance of HRQoL as a fundamental outcome domain in clinical trials of SLE [6]. Despite significant advancements in lupus therapy, patients continue to encounter considerable limitations in physical, mental, and social aspects of HRQoL [7]. Disease activity, alongside comorbidities, treatment complications, and organ damage, have been recognized as important factors that negatively impact HRQoL in SLE [8, 9]. Recently, we demonstrated a decline in self-reported physical health, increased fatigue, and impaired social functioning among SLE patients with high BMI, while the use of antimalarials and attainment of remission or low disease activity were all associated with favourable physical functioning [10–12].

Individuals living with SLE continue to experience considerable impairments in HRQoL compared with those with other chronic diseases, but not those with CNS-related diseases [13]. As expected, patients with neurological autoimmune disorders report greater fatigue and impaired HRQoL compared with the general population [14]. Patients with multiple sclerosis experience worse HRQoL compared with patients with IBD or RA [15]. Thus, we hypothesize that neuropsychiatric involvement contributes to poorer HRQoL within the SLE population.

Herein, we aimed to determine the impact of neuropsychiatric involvement in lupus patients on PROs within the framework of four phase III clinical trials. We further explored the frequencies of HRQoL outcomes in NPSLE patients with active *vs* inactive neuropsychiatric disease.

## Methods

### Study design and population

We conducted a post-hoc analysis using data from four double-blinded, placebo-controlled phase III trials of belimumab: BLISS-52 (NCT00424476) [16], BLISS-76 (NCT00410384) [17], BLISS-SC (NCT01484496) [18] and EMBRACE (NCT01632241) [19], which included 865, 819, 839 and 448 patients with SLE, respectively. All study participants were 18 years of age or older and met the classification criteria for SLE according to the revised ACR criteria [20].

In all trials, all patients were seropositive, which was defined as having an ANA titre of 1:80 or higher and/or an anti-dsDNA level of 30 IU/ml or higher. Serological assessments were performed at central laboratories (KingMed Diagnostics, International Biotech Island, Guangzhou, Guangdong, China, for Chinese sites; Q_2_ Solutions, formerly Quest Diagnostics, Valencia, CA, USA, for all other sites) using ELISA, except for ANA titres, which were determined using indirect IF on HEp-2 cells. Additionally, all participants had active SLE, as defined by a Safety of Estrogens in Lupus National Assessment (SELENA)-SLEDAI score of 6 or higher in BLISS-52 and BLISS-76, and a SELENA-SLEDAI score of 8 or higher in BLISS-SC and EMBRACE.

### Definition of NPSLE

For the purpose of the present study, the phenotype of SLE was categorized as either NPSLE or non-neuropsychiatric (non-NP) SLE at the baseline (week 0) assessment. The presence of NP involvement was determined based on the neuropsychiatric items of the classic BILAG index and/or the SLEDAI 2000 (SLEDAI-2K).

NP was defined as having a BILAG score of A, B, C or D in the neuropsychiatric domain and/or at least one neuropsychiatric descriptor scored in the SLEDAI-2K at baseline; this yielded 350 patients. Within the NPSLE group, the active NPSLE subgroup was identified as individuals with a score of A or B in the neuropsychiatric domain of BILAG and/or any neuropsychiatric descriptor scored in the SLEDAI-2K (*N* = 71). Accordingly, NPSLE patients with a BILAG score of C or D in the neuropsychiatric domain and no neuropsychiatric descriptor score in the SLEDAI-2K were categorized into the inactive NPSLE group; this yielded 269 participants with inactive NPSLE. The NPSLE group encompassed patients with a BILAG score of E in the neuropsychiatric domain of BILAG (*N* = 2618).

### Evaluation of HRQoL

The assessment of HRQoL in this study involved the use of three generic instruments. These included the Medical Outcomes Study Short Form 36 (SF-36) health survey [21], the Functional Assessment of Chronic Illness Therapy Fatigue (FACIT-F) scale [22] and the three-level version of the EQ-5D (EQ-5D-3L) [23]. The psychometric properties of SF-36 and FACIT-F have been thoroughly reviewed and recommended by the OMERACT SLE working group as secondary end points in clinical trials, indicating their suitability and reliability for assessing HRQoL in individuals with SLE [24].

The SF-36 comprises 36 questions that contribute to the calculation of scores for eight distinct subscales, each representing a specific aspect of HRQoL. These subscales encompass physical functioning (PF), role physical (RoleP), bodily pain (BP), general health (GH), social functioning (SocF), vitality (VT), role emotional (RE) and mental health (MH). The computation of scores for SF-36 subscales follows the guidelines outlined in the SF-36v2 manual [25]. Additionally, two summary scores are derived: the physical component summary (PCS) and the mental component summary (MCS). The physical aspects of SF-36 encompass PF, RoleP, BP and GH, whereas the mental aspects comprise SocF, VT, RE and MH. Higher scores in the SF-36 items indicate more favourable perceptions of HRQoL.

The evaluation of fatigue was carried out using the FACIT-F scale. The FACIT-F scale comprises 13 different items, and the responses provided by patients to these items are transformed into a score ranging from 0 (reflecting maximal fatigue) to 52 (representing minimal fatigue) [26].

The EQ-5D-3L health questionnaire is utilized to measure health status and comprises two components: a Visual Analogue Scale (VAS) and a descriptive part. The descriptive part includes questions related to five dimensions: mobility, self-care, usual activities, pain or discomfort, and anxiety or depression. Patients provide responses to these questions on a three-level response scale: no problems, some/moderate problems or extreme/major problems in each one of these dimensions. Following the guidelines provided in the EQ-5D-3L user guide [23], we defined Full Health State (FHS) as a response of ‘no problems’ in all five EQ-5D dimensions [27]. This combination of responses corresponds to an EQ-5D-3L utility index score of 1, indicating a state of full or perfect health.

### Statistical analysis

The data are presented in the form of numbers and percentages for categorical variables or means (s.d.) for normally distributed continuous variables. In the case of non-normal distributions, medians with interquartile ranges are provided. To compare unrelated continuous data, the Mann–Whitney *U* test was used. Associations between unrelated binomial variables were assessed using either the Pearson’s chi-squared (*χ*^2^) test or the Fisher’s exact test, depending on numbers of events after tabulation. Statistical significance was considered to be indicated by *P*-values of <0.05. All analyses were conducted using the R software version 4.1.0, developed by the R Foundation for Statistical Computing in Vienna, Austria.

### Ethics

GlaxoSmithKline (based in Uxbridge, UK) provided access to data from the BLISS trials through the Clinical Study Data Request consortium. The study adhered to the ethical principles outlined in the Declaration of Helsinki. Prior to their enrolment, written informed consent was obtained from all participants involved in the study. The study protocols for BLISS-52, BLISS-76, BLISS-SC and EMBRACE were approved by the regional ethics review boards for all participating centres, and the protocol for the present study was approved by the Swedish Ethical Review Authority under reference number 2019–05498.

## Results

### Demographics and clinical characteristics

The demographics of the NPSLE and non-NP SLE groups are presented in Supplementary Table S1, available at *Rheumatology* online. Despite the fact that severe active CNS involvement at the time of screening was an exclusion criterion by the trial protocols, we identified 71 patients with active NPSLE at baseline (week 0), among whom 16 patients exhibited severe NPSLE according to the trial protocol definition (seizures, psychosis, organic brain syndrome, cerebrovascular accident, cerebritis, or CNS vasculitis). The majority of the participants were women (94.6%). The mean (s.d.) age of study participants was at baseline 38.2 years (11.7). The NPSLE and non-NP SLE groups did not differ with regard to sex, extra-neuropsychiatric disease activity or irreversible organ damage, but patients with NPSLE were older than non-NP SLE individuals [40.9 (11.0) *vs* 37.8 (11.8); *P* *<* 0.001]. Moreover, NPSLE patients were more likely to be White/Caucasians and less likely to be Asians. The neuropsychiatric manifestations are summarized in Table 1, with lupus headache, cranial neuropathies, and peripheral neuropathies being the most common. Psychosis, acute confusional state (ACS), and cranial neuropathies were the most common neuropsychiatric manifestations in the active NPSLE group. It is worth noting that 6 patients with active NPSLE (headache: *n* = 4; psychosis: *n* = 1; ACS: *n* = 1) were only captured by the BILAG scoring system.

**Table 1.** Neuropsychiatric events in the entire NPSLE population

	All NPSLE patients (*N* = 350)	Inactive NPSLE (*N* = 279)	Active NPSLE (*N* = 71)	*P* value
**Neuropsychiatric events**				
Transverse myelitis; *n* (%)	1 (0.3)	1 (0.4)	0 (0.0)	1.000
Aseptic meningitis; n (%)	0 (0.0)	0 (0.0)	0 (0.0)	NA
Movement disorder; *n* (%)	8 (2.3)	3 (1.1)	5 (7.0)	0.035*
Mononeuritis multiplex; *n* (%)	6 (1.7)	6 (2.2)	0 (0.0)	0.463
Psychosis; *n* (%)	4 (1.1)	0 (0.0)	4 (5.6)	0.001*
Seizures; *n* (%)	0 (0.0)	0 (0.0)	0 (0.0)	NA
Depression, *n* (%)	52 (14.9)	39 (14.0)	13 (18.3)	0.466
Stroke; *n* (%)	2 (0.6)	1 (0.4)	1 (1.4)	0.868
Cranial neuropathy; *n* (%)	7 (2.0)	0 (0.0)	7 (9.9)	<0.001*
Optic neuritis; *n* (%)	11 (3.1)	0 (0.0)	11 (15.5)	<0.001*
Headache; *n* (%)	208 (59.4)	165 (59.1)	43 (60.6)	0.934
Acute confusional state; *n* (%)	17 (4.9)	6 (2.2)	11 (15.5)	<0.001*
Polyneuropathy; *n* (%)	72 (20.6)	51 (18.3)	21 (29.6)	0.053

Data are presented as numbers (percentage).

* Statistically significant *P* value. NA: not applicable.

### Neuropsychiatric involvement in SLE is associated with impaired quality of life

**Figure 1.**
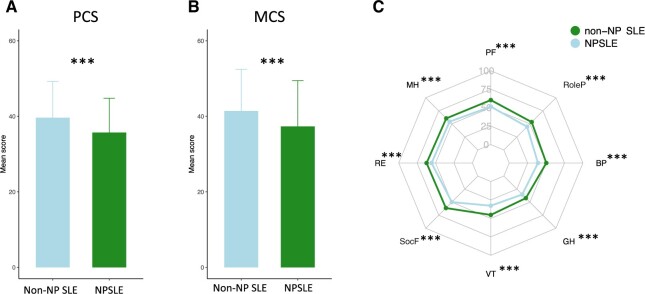
Comparisons of HRQoL between NPSLE and non-neuropsychiatric (non-NP) SLE patients. The figure illustrates the comparison of 36-item Short Form health survey questionnaire (SF-36) scores between NPSLE (green) and non-NP SLE (light blue) patients. The bar chart illustrates comparisons of SF-36 component summary scores for (**A**) the physical component summary (PCS) and (**B**) the mental component summary (MCS). (**C**) The radial chart illustrates comparisons of mean SF-36 subscale scores for physical functioning (PF), role physical (RoleP), bodily pain (BP), general health (GH), vitality (VT), social functioning (SocF), role emotional (RE), and mental health (MH). Asterisks indicate statistically significant associations: *P *<* 0.05, **P *<* 0.01, ***P *<* 0.001. Data are presented as the mean (s.d.). HRQoL: health-related quality of life

Patients with NPSLE reported lower SF-36 PCS [35.7 (9.1) *vs* 39.6 (9.6); *P* *<* 0.001] and SF-36 MCS scores [37.3 (12.1) *vs* 41.4 (11.0); *P* *<* 0.001] compared with non-NP SLE patients (Fig. 1A, B). Importantly, significant differences were seen in all SF-36 subscales i.e. PF [51.3 (24.8) *vs* 60.3 (25.5); *P* *<* 0.001], RoleP [44.6 (27.4) *vs* 53.5 (27.0); *P* *<* 0.001], BP [39.0 (20.4) *vs* 50.3 (24.5); *P* *<* 0.001], GH [35.3 (18.2) *vs* 42.2 (18.9); *P* *<* 0.001], VT [32.8 (21.3) *vs* 45.3 (21.3); *P* *<* 0.001], SocF [49.9 (25.3) *vs* 61.2 (49.9); *P* *<* 0.001], RE [55.4 (28.8) *vs* 62.2 (26.9); *P* *<* 0.001] and MH [54.2 (21.3) *vs* 60.5 (19.4); *P* *<* 0.001] (Fig. 1C).

**Figure 2.**
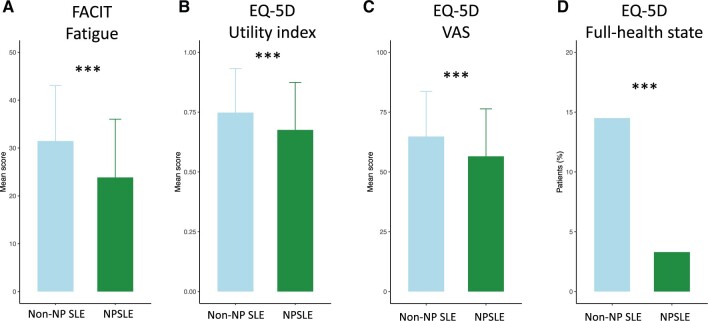
Comparisons of HRQoL between NPSLE and non-neuropsychiatric (non-NP) SLE patients. The figure illustrates the comparison of HRQoL perceptions between SLE patients with (green) and without (light blue) NP with regard to (**A**) FACIT-F scores, (**B**) EQ-5D utility index scores, (**C**) EQ-VAS scores and (**D**) EQ-5D FHS. Asterisks indicate statistically significant associations: *P *<* 0.05, **P *<* 0.01, ***P *<* 0.001. Data are presented as the mean (s.d.). HRQoL: health-related quality of life; FACIT-F: Functional Assessment of Chronic Illness Therapy—Fatigue; EQ-5D: EuroQol research foundation 5-dimension; VAS: visual analogue scale; FHS: full health state

Compared with patients with non-NP SLE, patients with NPSLE exhibited greater FACIT-F scores [23.8 (12.2) *vs* 31.5 (11.6); *P* *<* 0.001] (Fig. 2A). Additionally, patients with NPSLE reported lower EQ-VAS scores [56.5 (19.9)] compared with patients without NP [64.8 (18.8); *P* *<* 0.001], while NPSLE patients reported worse EQ-5D utility index scores [0.68 (0.20)] than non-NP SLE patients [0.75 (0.18); *P* *<* 0.001] (Fig. 2B, C). Finally, the proportion of patients experiencing FHS was lower within the NPSLE group [3.3% *vs* 14.5%; odds ratio (OR): 0.28; 95% CI: 0.14–0.57; *P* *<* 0.001; Fig. 2D).

**Figure 3.**
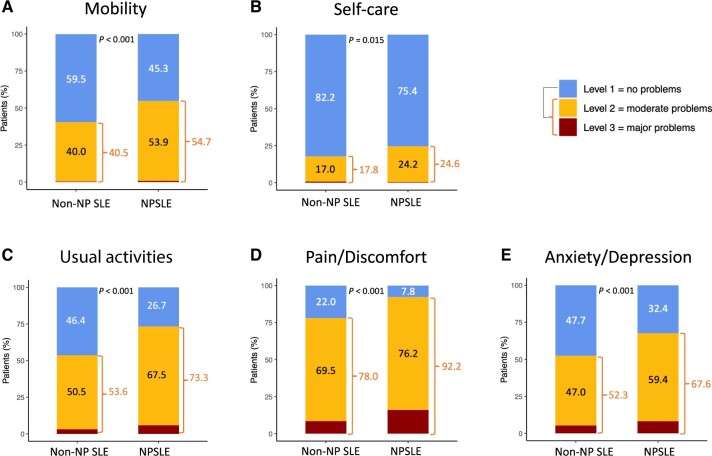
EQ-5D dimensions in NPSLE and non-neuropsychiatric (non-NP) SLE patients. The figure illustrates comparisons between NPSLE and non-NP SLE patients with regard to the five different dimensions of the EQ-5D questionnaire i.e. (**A**) mobility, (**B**) self-care, (**C**) usual activities, (**D**) pain/discomfort and (**E**) anxiety/depression. The proportions of patients reporting no problems (level 1), moderate problems (level 2) and major problems (level 3) are denoted by colour-coded sections (blue, yellow and red, respectively). *P*-values derived from Pearson’s χ^2^ tests indicate comparisons of level 1 (no problems) experienced between patients with and without NP

Next, we investigated differences in the various dimensions of the EQ-5D questionnaire separately between the NPSLE and the non-NP SLE groups, in terms of reports of no problems *vs* moderate or major problems. A lower proportion of NPSLE patients reported no problems in all dimensions, i.e. mobility (45.3% *vs* 59.5%; OR: 0.45; 95% CI: 0.36–0.57; *P* *<* 0.001), self-care (75.4% *vs* 82.2%; OR: 0.51; 95% CI: 0.38–0.69; *P* *=* 0.015), usual activities (26.7% *vs* 46.4%; OR: 0.39; 95% CI: 0.31–0.49; *P* *<* 0.001), pain/discomfort (7.8% *vs* 22%; OR: 0.4; 95% CI: 0.32–0.51; *P* *<* 0.001) and anxiety/depression (32.4% *vs* 47.7%; OR: 0.44; 95% CI: 0.35–0.55; *P* *<* 0.001) (Fig. 3).

### Impaired HRQoL in NPSLE persists regardless of neuropsychiatric disease activity

**Figure 4.**
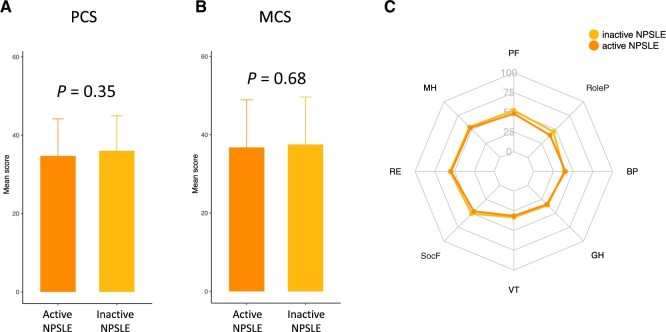
Comparisons of HRQoL between patients with active NPSLE and patients with prior yet currently quiescent NPSLE. The figure illustrates comparisons of 36-item Short Form health survey questionnaire (SF-36) scores between patients with active NPSLE (orange) and patients with prior yet currently quiescent NPSLE (yellow). The bar chart illustrates comparisons of SF-36 component summary scores for (**A**) the physical component summary (PCS) and (**B**) the mental component summary (MCS). (**C**) The radial chart illustrates comparisons of mean SF-36 subscale scores for physical functioning (PF), role physical (RoleP), bodily pain (BP), general health (GH), vitality (VT), social functioning (SocF), role emotional (RE), and mental health (MH). Asterisks indicate statistically significant associations: *P *<* 0.05, **P *<* 0.01, ***P *<* 0.001. Data are presented as the mean (s.d.). HRQoL: health-related quality of life

**Figure 5.**
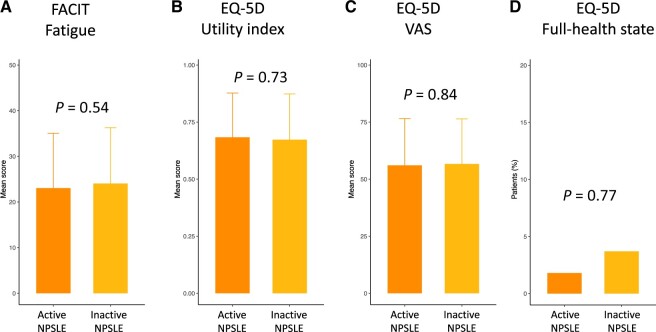
Comparisons of HRQoL between active and inactive NPSLE. The figure illustrates comparisons of HRQoL perceptions between patients with active (orange) and patients with prior yet currently inactive (yellow) neuropsychiatric involvement with regard to (**A**) FACIT-F scores, (**B**) EQ-5D utility index scores, (**C**) EQ-VAS scores and (**D**) EQ-5D FHS. Asterisks indicate statistically significant associations: *P *<* 0.05, **P *<* 0.01, ***P *<* 0.001. Data are presented as the mean (s.d.). HRQoL: health-related quality of life; FACIT-F: Functional Assessment of Chronic Illness Therapy—Fatigue; EQ-5D: EuroQol research foundation 5-dimension; VAS: visual analogue scale; FHS: full health state

We compared patients with active NPSLE at baseline against those with prior yet currently inactive NPSLE. We observed no statistically significant differences in SF-36 PCS or MCS scores, or any of the SF-36 subscales (Fig. 4). Furthermore, no differences were seen in the FACIT-F, EQ-VAS or EQ-5D utility index scores, or EQ-5D FHS frequencies (Fig. 5).

## Discussion

In the present study, we used baseline data from four RCTs to investigate the impact of NP on SLE patients’ HRQoL. We showed that patients with NPSLE experienced a more impaired HRQoL and more fatigue compared with SLE patients without NP. More importantly, we demonstrated that SLE patients with active and patients with prior yet currently inactive neuropsychiatric disease exhibit comparable experiences of HRQoL and fatigue, suggesting that HRQoL impairments in NPSLE persist even after the resolution of the neuropsychiatric manifestations.

SLE is inherently linked with compromised HRQoL [28]. Over the past few decades, several studies have been undertaken to elucidate the factors that contribute to the HRQoL impairments among patients with SLE, as well as factors that contribute to alleviation of those impairments. Importantly, attainment of remission or Lupus Low Disease Activity State (LLDAS) have been shown to enhance overall HRQoL [11]. Furthermore, medications that effectively diminish disease activity in SLE have a positive impact on HRQoL. Specifically, use of antimalarial agents has been shown to enhance physical functioning in SLE, and the targeted biologics belimumab and anifrolumab have also been shown to exert favourable effects [12, 29–31]. Distinct disease manifestations have been shown to be associated with poor HRQoL. As anticipated, musculoskeletal manifestations have been associated with impaired physical and mental functioning [32], while LN has been associated with severe fatigue. Our study suggests that neuropsychiatric involvement also constitutes an important contributing factor to HRQoL impairments in SLE. To this end, it is essential to acknowledge that SLE patients’ HRQoL experience is not solely attributed to disease-related factors.

To date, the impact of neuropsychiatric involvement on SLE patients’ HRQoL has been mainly explored by means of SF-36 assessments in two cohort studies [4, 33]. Monahan *et al.* demonstrated that individuals with SLE who exhibit neuropsychiatric symptoms experience a significant reduction in their HRQoL, both compared with the general population and compared with individuals with other chronic diseases [33]. Data from the same cohort revealed that objective cognitive impairment is a prevalent symptom in SLE populations; however, its impact on patients’ HRQoL is unclear. Cohort studies from the SLICC collaboration indicate that patients with active neuropsychiatric disease display lower SF-36 component summary scores compared with patients without NPSLE, as well as compared with those with resolved neuropsychiatric events [4]. Notably, SLE patients with neuropsychiatric events exhibited worse HRQoL experiences irrespective of the attribution of these events [34]. Numerous follow-up studies have been conducted within the SLICC collaboration, focusing on specific neuropsychiatric manifestations and evaluations of HRQoL. Patients with cerebrovascular events reported a persistent decline in HRQoL, although the neurological deficits completely resolved or improved in the majority of cases [35]. By contrast, seizures did not have a notable impact on scores of either the mental or the PCS [36]. Most cases of psychosis and neuropathies exhibited resolution or improvement over time, and this positive trend was linked to improvement in SF-36 component summary scores [37]. As expected, mood disorders were shown to have a pronounced negative impact on the mental component of SF-36 [38], while both mental and PCS scores were significantly reduced in patients with headache compared with those without [39]. In our study, most neuropsychiatric events consisted of neuronopathies, headaches, and mood disorders. We were able to corroborate SF-36 findings from the existing literature with regard to these specific manifestations and, by addressing our research questions using multiple PROMs, further advance the understanding of how NPSLE impacts various aspects of patients’ HRQoL.

The concept of EQ-5D FHS has gained attention as a viable PROM in SLE clinical trials and observational studies, owing to recent studies that unveiled strong psychometric properties in SLE populations [27, 40, 41]. In comparison with an age- and sex-matched general US population, individuals with SLE less frequently report FHS [13]. Female sex, higher disease activity, and increasing damage accrual have been shown to hamper FHS experience in SLE [41]. Nevertheless, there is a scarcity of studies that explore how disease-related manifestations may impact the attainment of FHS in patients with SLE. In the present study, we demonstrated that SLE patients with current or prior NP had a lower likelihood of experiencing FHS.

HRQoL aspects are commonly disregarded in routine clinical practice, yet their significance is progressively garnering recognition [28]. Accordingly, updated recommendations for the management of SLE highlight the importance of HRQoL as an integral component of the evaluation of the disease that should be considered in treatment decisions [42]. In clinical practice, physicians have a multitude of tests and assessment instruments at their disposal, allowing them to select the most appropriate ones based on the individual patient’s needs. It is important to note that NPSLE patients represent a distinct subgroup of the SLE population, often suffering from depression and other psychiatric conditions. Additionally, the neurological manifestations may lead to functional impairment, which highlights the need for PROMs capturing the full spectrum of HRQoL. However, incorporation of these instruments into clinical practice for improved management of NPSLE needs support from robust evidence [28]. To this end, it is evident that achieving remission and ensuring a state of adequate HRQoL for individuals with NPSLE cannot be solely addressed through pharmacological treatment. NPSLE is a challenging condition to treat, necessitating intensive immunosuppressant therapy, often for a long time, which is associated with toxicities, thus impacting HRQoL in a multifaceted manner. With this background, it becomes apparent that structured non-pharmacological strategies are also needed to support improvements in aspects of HRQoL [43].

Fatigue is a major complaint for patients with SLE and is closely linked to the experience of depression and anxiety. SLE activity and the administration of glucocorticoids have been shown to be independently associated with fatigue [44]. To our knowledge, this is the first study demonstrating a clear association between NP and heightened fatigue levels in patients with SLE. It is worth emphasizing that uniform treatment approaches against fatigue do not exist for rheumatic diseases. While one could expect that improved disease management might alleviate fatigue, it is important to acknowledge the intricate nature of the underlying causes of fatigue [44]. Recent studies have suggested that the activation of microglia cells is associated with a chronic fatigue syndrome [45], which is intriguing in light of microglia activation being considered an early pathogenetic mechanism in NPSLE [46]. Achieving remission or clinically meaningful treatment responses does not guarantee the absence of fatigue, as evidenced in recent research [47]. Murine studies have shown that attenuation of systemic inflammation does not restore the microglia phenotype and does not always improve the neuropsychiatric disease [48]. From a clinical standpoint, a recent study showed that anxiety and depression persist in SLE despite the attainment of remission or low disease activity [49]. While it is crucial to stress that effective disease control remains imperative for mitigating the component of fatigue that is driven by inflammation, effective management of fatigue in SLE should incorporate a comprehensive and holistic approach to patient care [43, 50]. To address this, the development of more robust and user-friendly measurement tools, along with increased awareness among both clinicians and patients regarding the multifaceted nature of fatigue, may result in improved fatigue management [44].

Being a post-hoc analysis of clinical trial data, our study is subject to certain limitations inherent in its design. Importantly, the BLISS trial protocols excluded patients with active CNS vasculitis and severe NPSLE (e.g. seizures). Consequently, the results should not be generalized to severe active forms of NPSLE. The prevalence of headache in the study may be considered high. Importantly, reliable data on the prevalence of active lupus headache may be obtained by the BILAG item ‘severe unremitting lupus headache’ contributing to a BILAG score of B in the neuropsychiatric domain, herein yielding 43 cases only, which is reassuring. By contrast, the high prevalence of headache in the entire NPSLE population was driven by scoring resulting in BILAG C in the neuropsychiatric domain. Additionally, data on some potential confounding factors were unavailable, including FM as a comorbid condition, socio-economic status, or information regarding daily physical activity. A major strength of our methodology lies in the incorporation of three different PROMs i.e. SF-36, EQ-5D and FACIT-F, providing a comprehensive characterization of HRQoL aspects in this SLE population and enhancing the overall robustness of the investigation.

NP in patients with SLE not only contributes to organ damage accrual and increased morbidity and mortality but also exerts a negative impact on HRQoL and the degree of fatigue [2]. This underscores the importance of holistic management for NPSLE, including early interventions tailored to this specific patient group towards improvement of long-term HRQoL outcomes. Development of novel pharmacological and non-pharmacological approaches aimed at desirable HRQoL experiences in people living with SLE remains a need and should be incorporated in the research agenda for the management of SLE.

## Supplementary Material

keae216_Supplementary_Data

## Data Availability

The datasets used and analysed during the present study can be made available through the Clinical Study Data Request consortium.
