# Supplementary material for: Patients with NPSLE experience poorer HRQoL and more fatigue than SLE patients with no neuropsychiatric involvement, irrespective of neuropsychiatric activity
Source: Rheumatology (Oxford). 2024 Apr 5;63(9):2494–502. doi: 10.1093/rheumatology/keae216 (PMC11403281; doi:10.1093/rheumatology/keae216)
Supplement: keae216_Supplementary_Data [file keae216_supplementary_data.pdf]

Supplementary Table S1. Demographics and clinical features of SLE patients in the pooled study population.

	All patients (N = 2968)	Non-NP SLE (N = 2618)	NPSLE (N = 350)	*p* value
**Demographics**				
Age; mean (S.D.)	38.17 (11.73)	37.80 (11.78)	40.89 (10.98)	**< 0.001**
Female sex; n (%)	2808 (94.6)	2476 (94.6)	332 (94.9)	0.926
Ethnicity; n (%)				
Asian	537 (18.1)	509 (19.4)	28 (8.0)	**< 0.001**
Black/African American	680 (22.9)	604 (23.1)	76 (21.7)	0.617
Indigenous American*	451 (15.2)	406 (15.5)	45 (12.9)	0.223
White/Caucasian	1300 (43.8)	1099 (42.0)	201 (57.4)	**< 0.001**
**Clinical features**				
SLE disease duration; mean (S.D.)	6.53 (6.57)	6.39 (6.51)	7.58 (6.98)	**0.002**
SDI score; mean (S.D.)	0.71 (1.16)	0.62 (1.06)	1.37 (1.55)	**< 0.001**
Extra-NP SDI score; mean (S.D.)	0.66 (1.15)	0.60 (1.03)	0.96 (1.24)	**< 0.01**
SLEDAI-2K; mean (S.D.)	10.24 (3.64)	10.11 (3.49)	11.25 (4.48)	**< 0.001**
Non-NP SLEDAI-2K; mean (S.D.)	10.08 (3.56)	10.11 (3.49)	9.88 (4.04)	0.273
Clinical SLEDAI-2K; mean (S.D.)	7.78 (3.45)	7.60 (3.28)	9.13 (4.26)	**< 0.001**
Non-NP Clinical SLEDAI-2K; mean (S.D.)	7.62 (3.33)	7.60 (3.28)	7.76 (3.69)	0.412

Data are presented as numbers (percentage) or means (standard deviation). Statistically significant *p* values are in bold.

*Alaska Native or American Indian from North, South or Central America.

NA: not applicable; NP: neuropsychiatric; NPSLE: neuropsychiatric systemic lupus erythematosus; S.D.: standard deviation; SDI: Systemic Lupus International Collaborating Clinics (SLICC)/American College of Rheumatology (ACR) Damage Index; SLE: systemic lupus erythematosus; SLEDAI-2K: Systemic Lupus Erythematosus Disease Activity Index 2000.
